# Supplementary figures and images for: Structure and mechanistic features of the prokaryotic minimal RNase P
Source: eLife. 2021 Jun 28;10:e70160. doi: 10.7554/eLife.70160 (PMC8266387; doi:10.7554/eLife.70160)

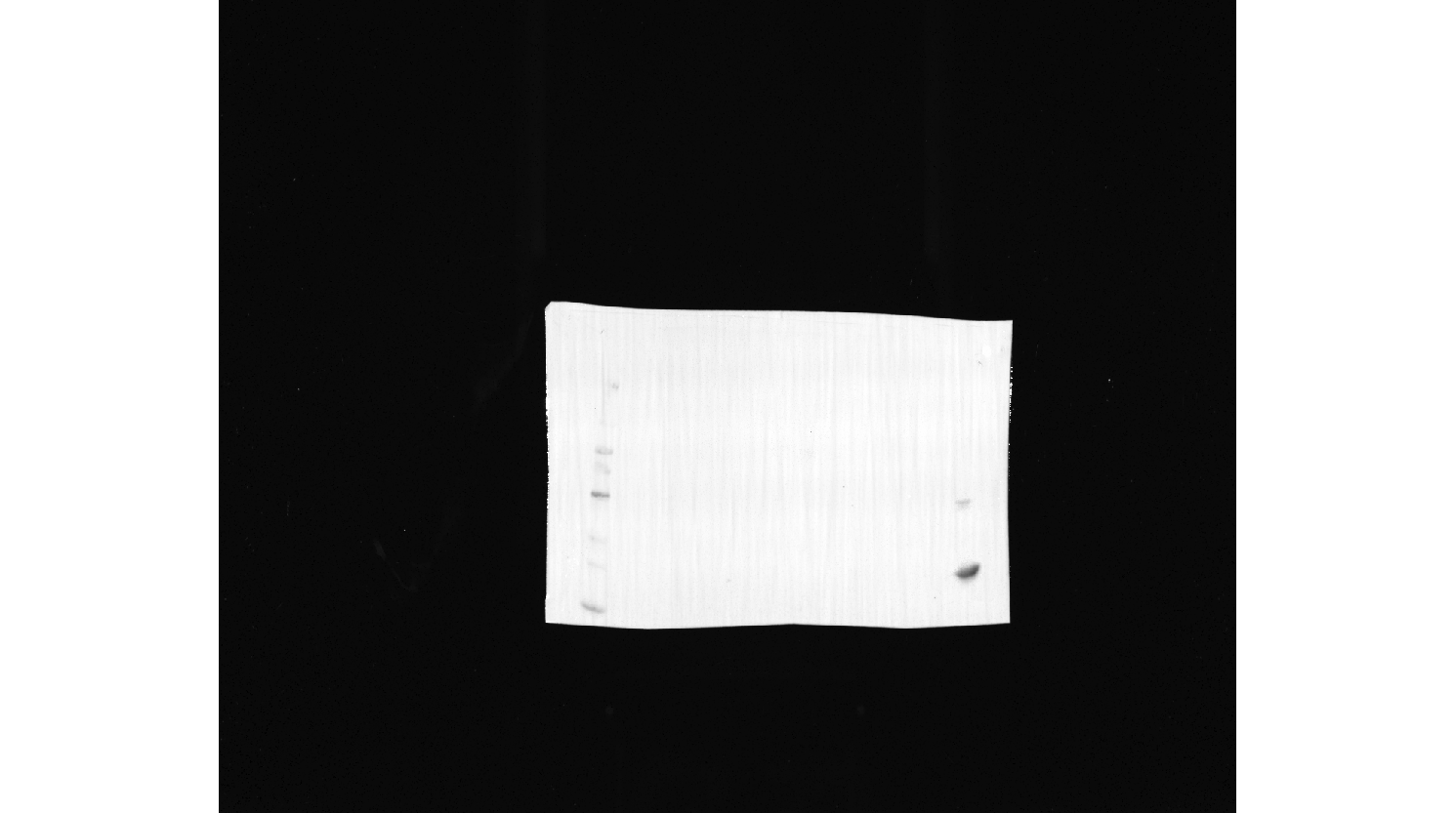

Supplement: Figure 1—figure supplement 1—source data 1. [file elife-70160-fig1-figsupp1-data1.zip › Figure 1-figure supplement 1-source data 1/Western Blot_MonoQ_Hhal_raw.tif]

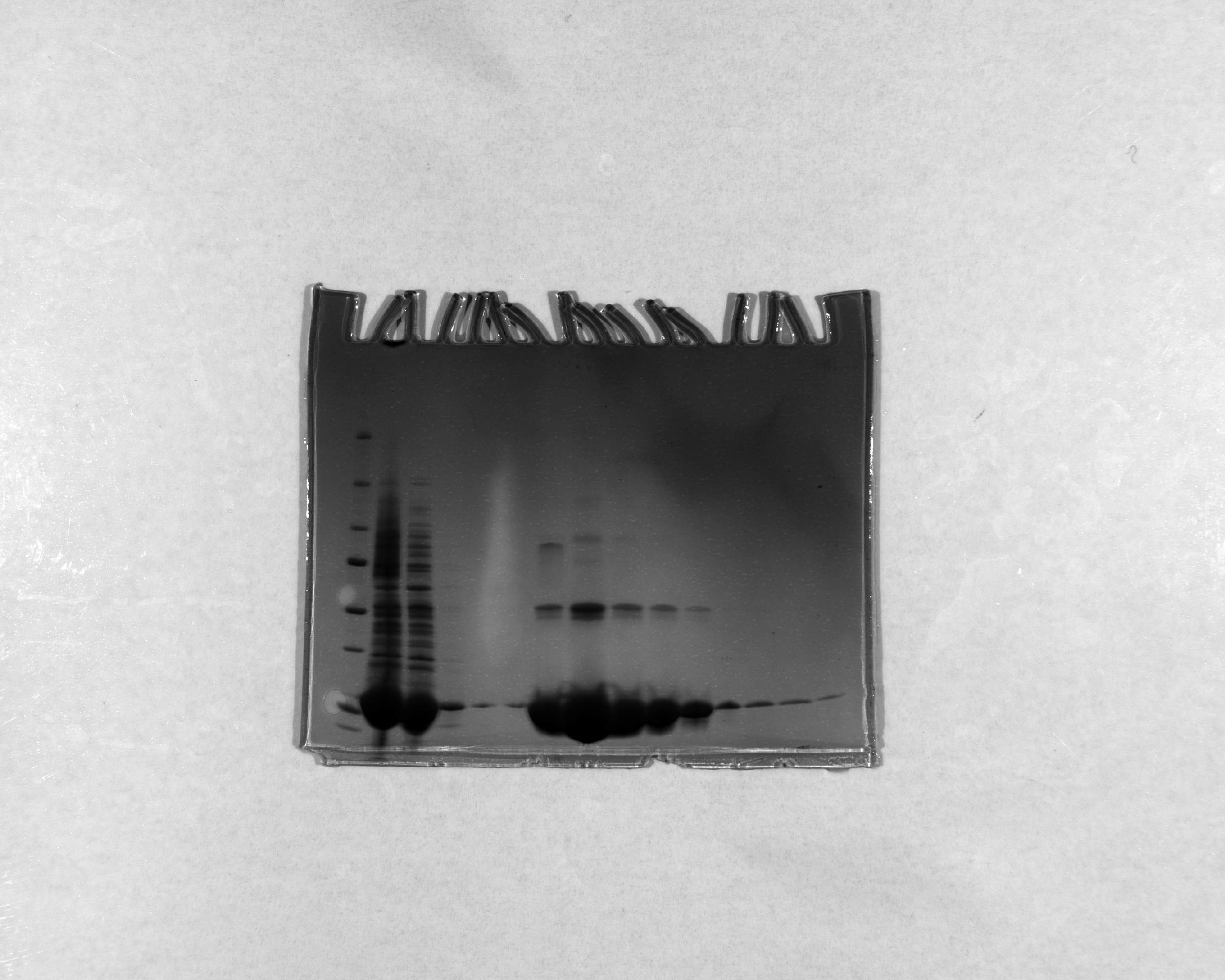

Supplement: Figure 1—figure supplement 1—source data 1. [file elife-70160-fig1-figsupp1-data1.zip › Figure 1-figure supplement 1-source data 1/Coomassie_PAGE_HisTrap_Hhal_raw.tif]

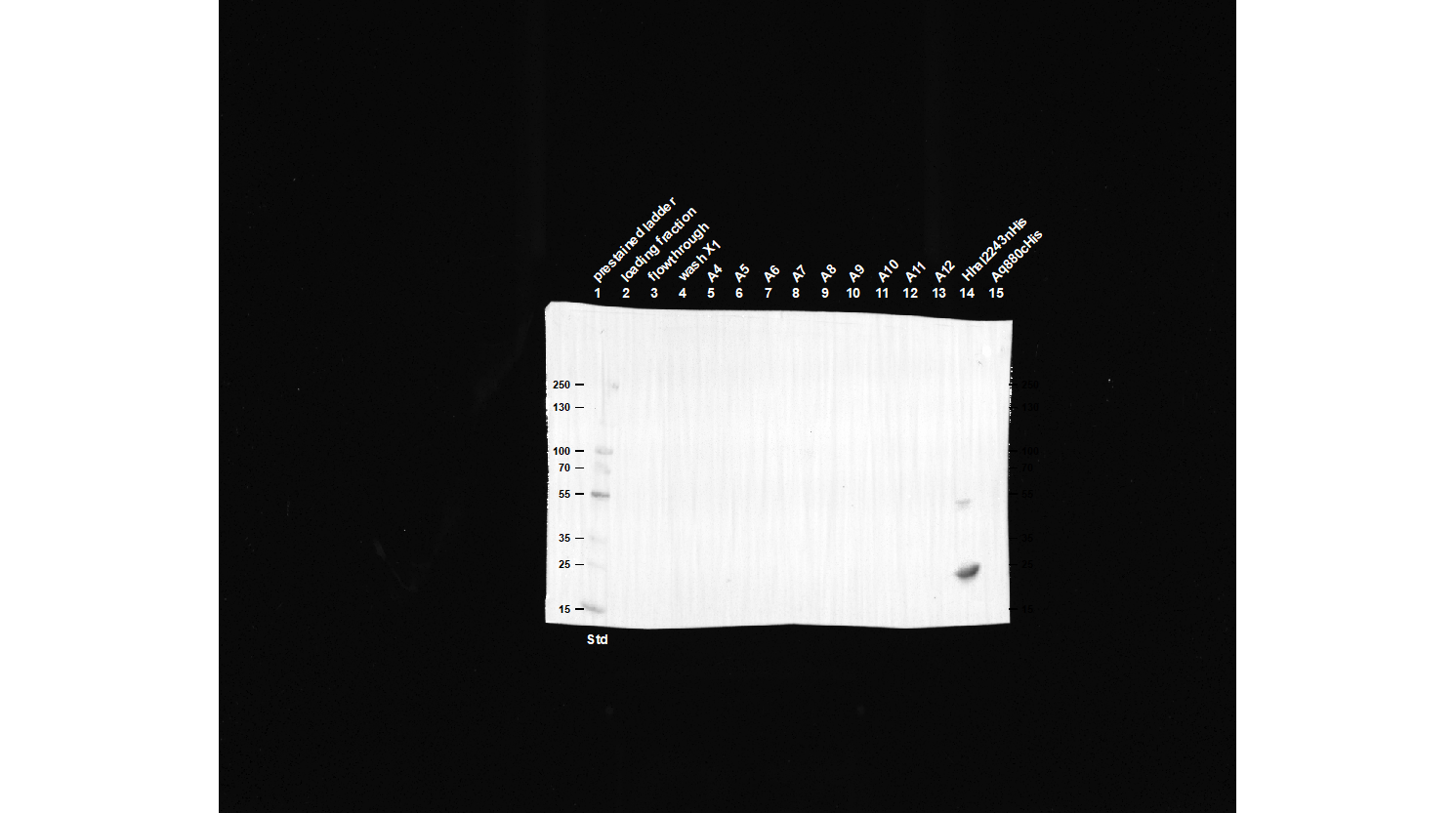

Supplement: Figure 1—figure supplement 1—source data 1. [file elife-70160-fig1-figsupp1-data1.zip › Figure 1-figure supplement 1-source data 1/Western_Blot_MonoQ_Hhal_labeled.tif]

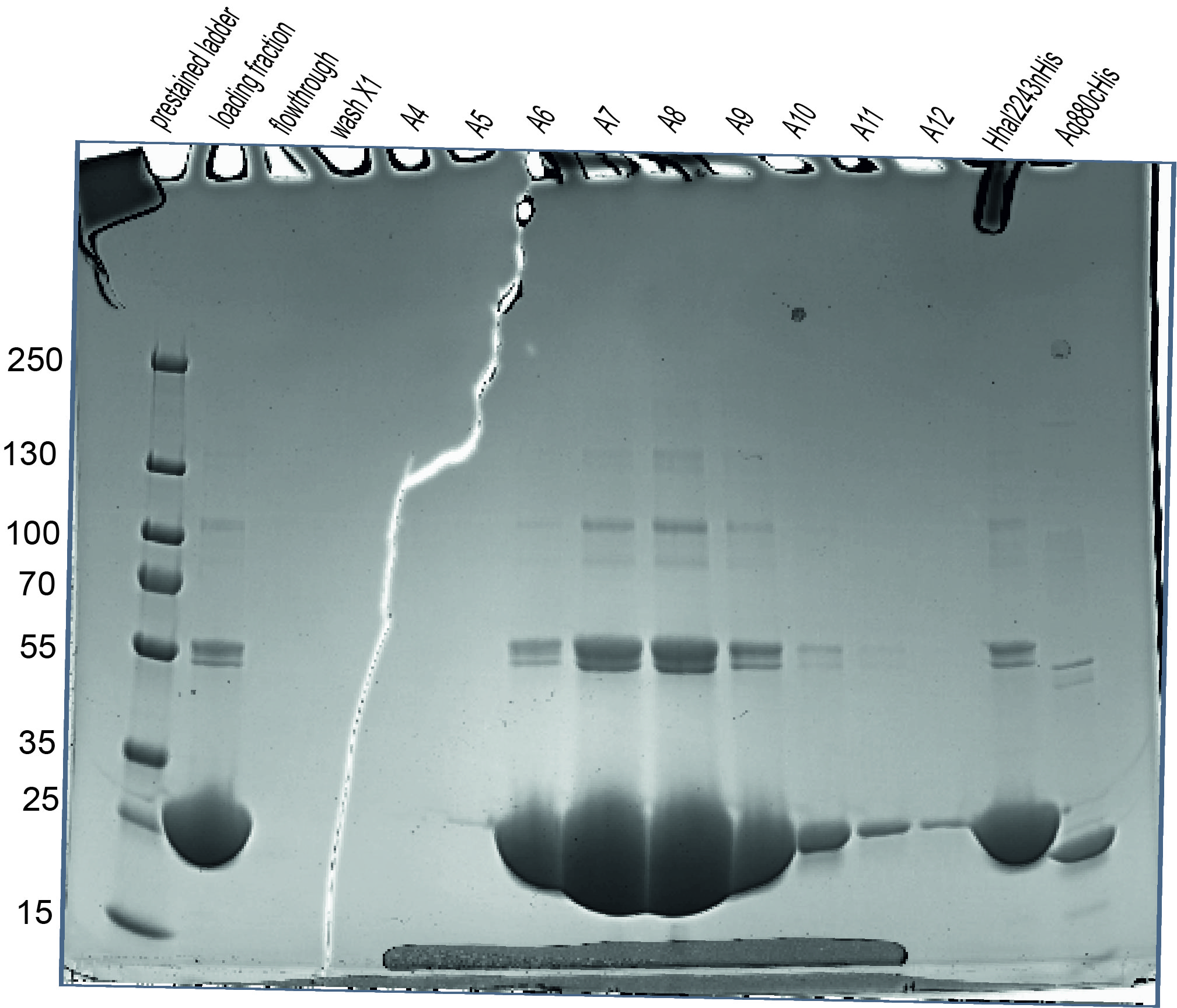

Supplement: Figure 1—figure supplement 1—source data 1. [file elife-70160-fig1-figsupp1-data1.zip › Figure 1-figure supplement 1-source data 1/Coomassie_PAGE_MonoQ_Hhal_labeled.jpg]

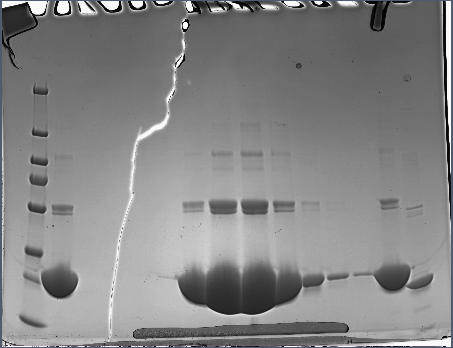

Supplement: Figure 1—figure supplement 1—source data 1. [file elife-70160-fig1-figsupp1-data1.zip › Figure 1-figure supplement 1-source data 1/Coomassie_PAGE_MonoQ_Hhal_raw.tif]

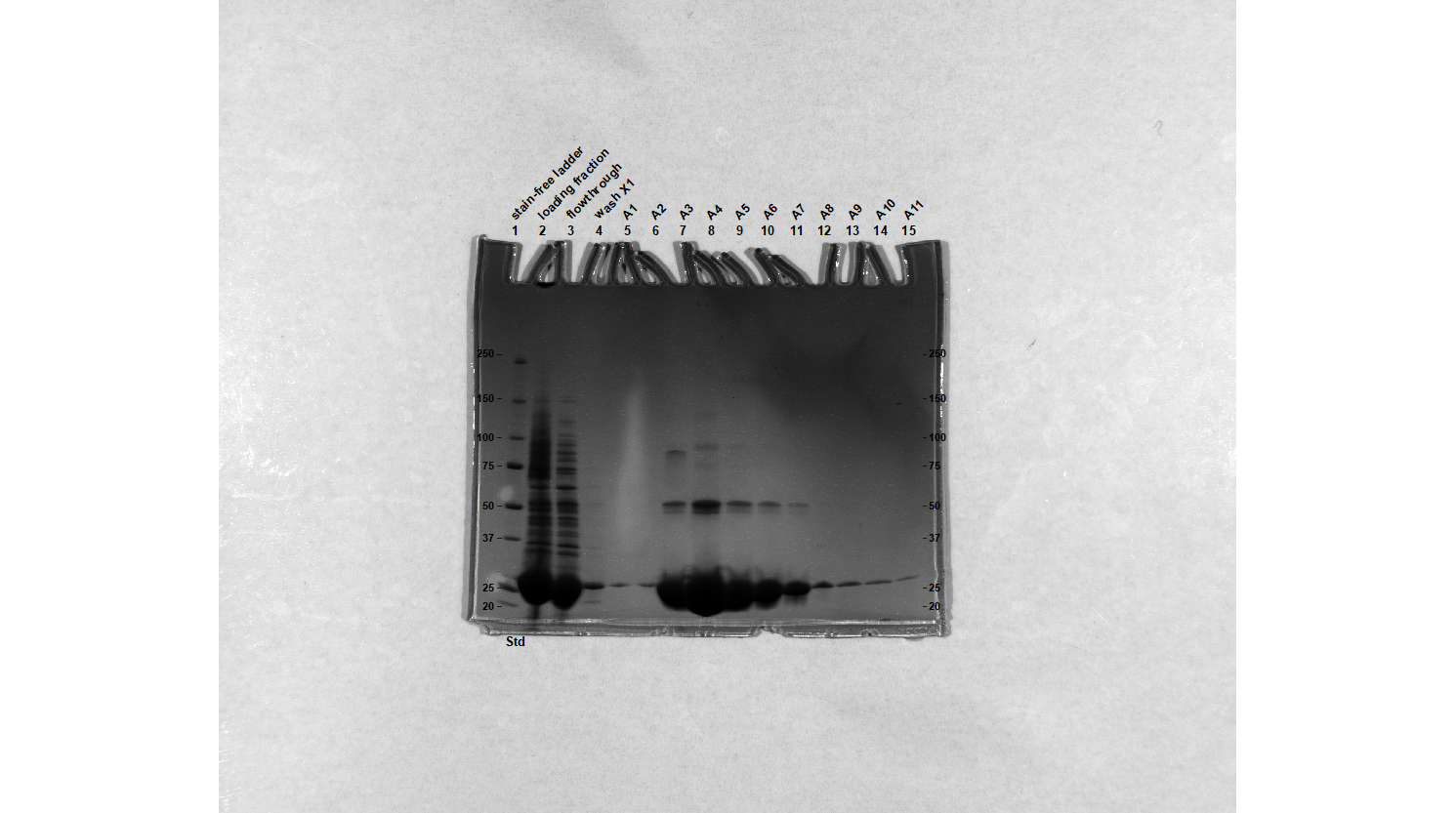

Supplement: Figure 1—figure supplement 1—source data 1. [file elife-70160-fig1-figsupp1-data1.zip › Figure 1-figure supplement 1-source data 1/Coomassie_PAGE_HisTrap_Hhal_labeled.tif]

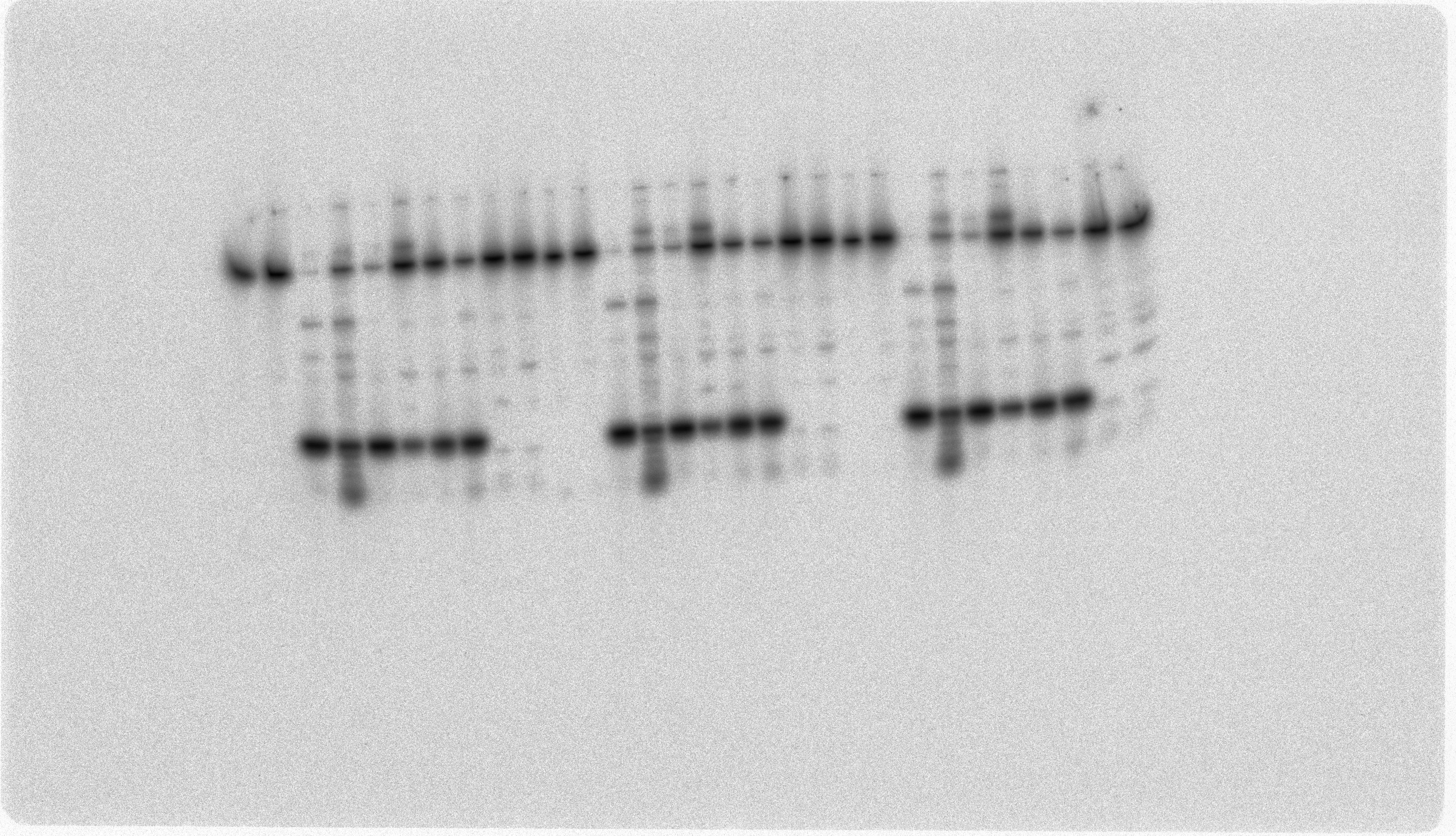

Supplement: Figure 1—figure supplement 2—source data 1. [file elife-70160-fig1-figsupp2-data1.zip › Figure 1-figure supplement 2-source data 1/Aq_Hhal_Mg_Mn_Sr_Ca_raw.jpg]

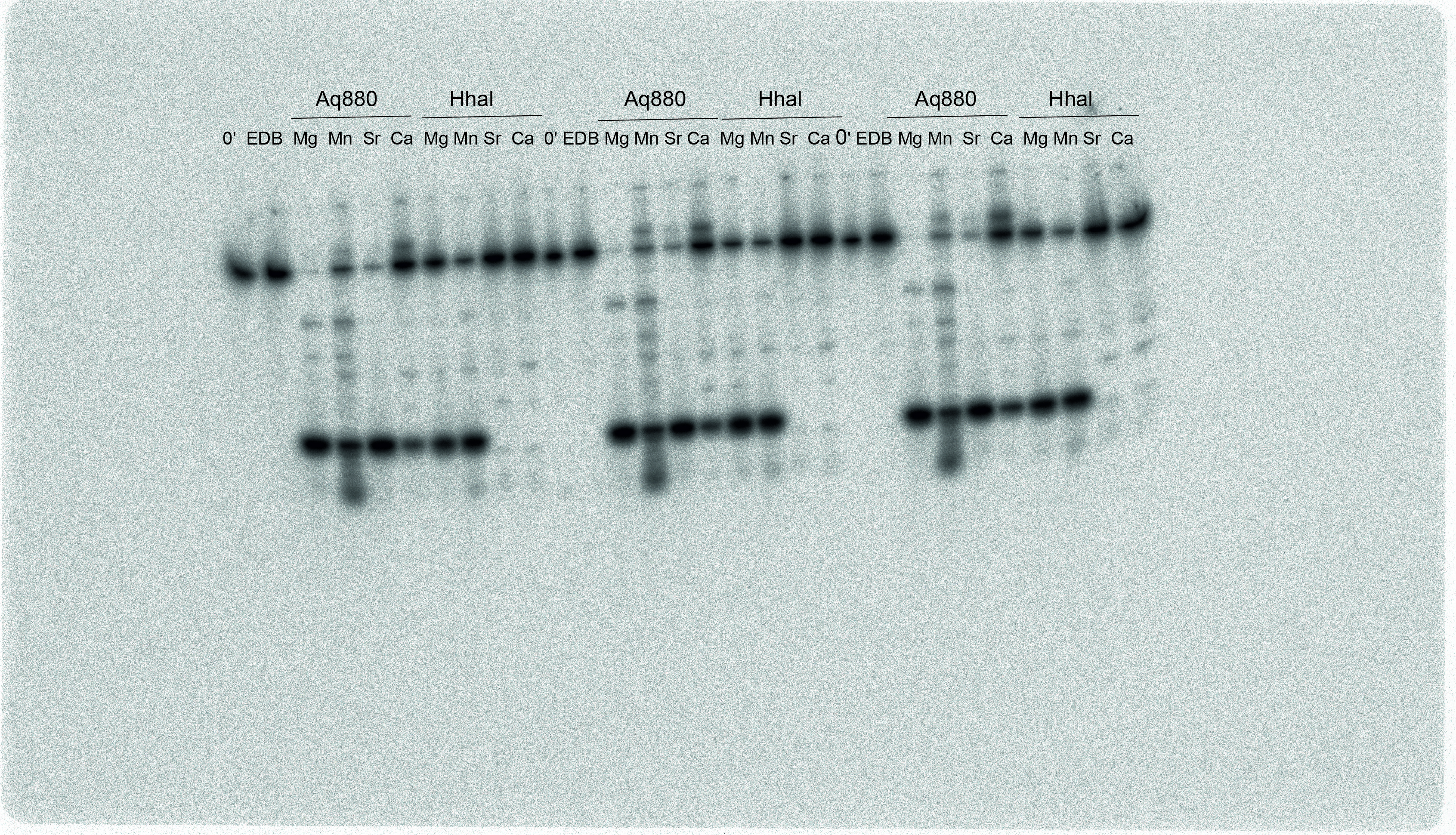

Supplement: Figure 1—figure supplement 2—source data 1. [file elife-70160-fig1-figsupp2-data1.zip › Figure 1-figure supplement 2-source data 1/Aq_Hhal_Mg_Mn_Sr_Ca_labeled.jpg]

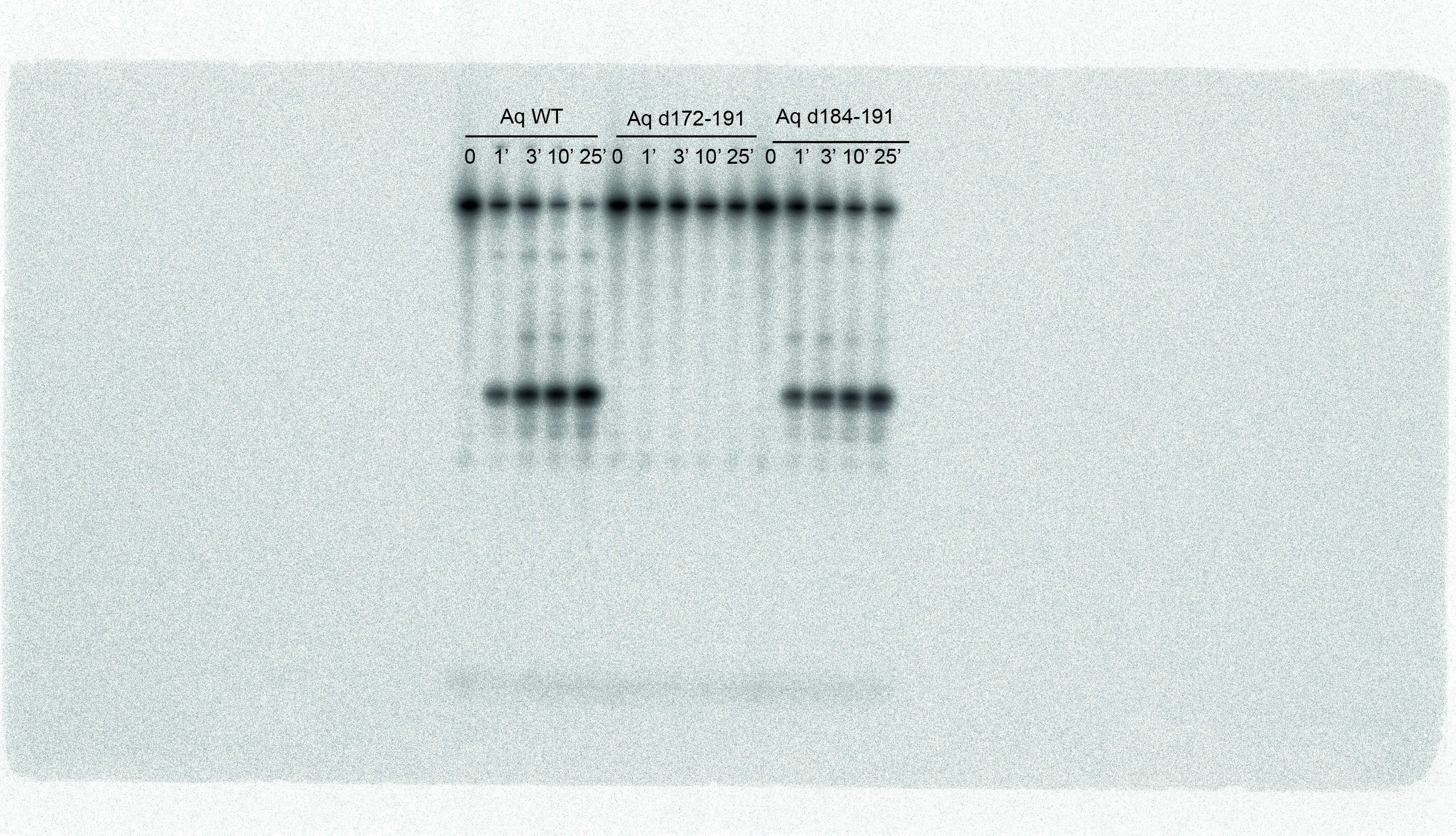

Supplement: Figure 2—source data 1. [file elife-70160-fig2-data1.zip › Figure_2_source-data_1/Aq_wt d172-191_d184-191 50nM_labeled.jpg]

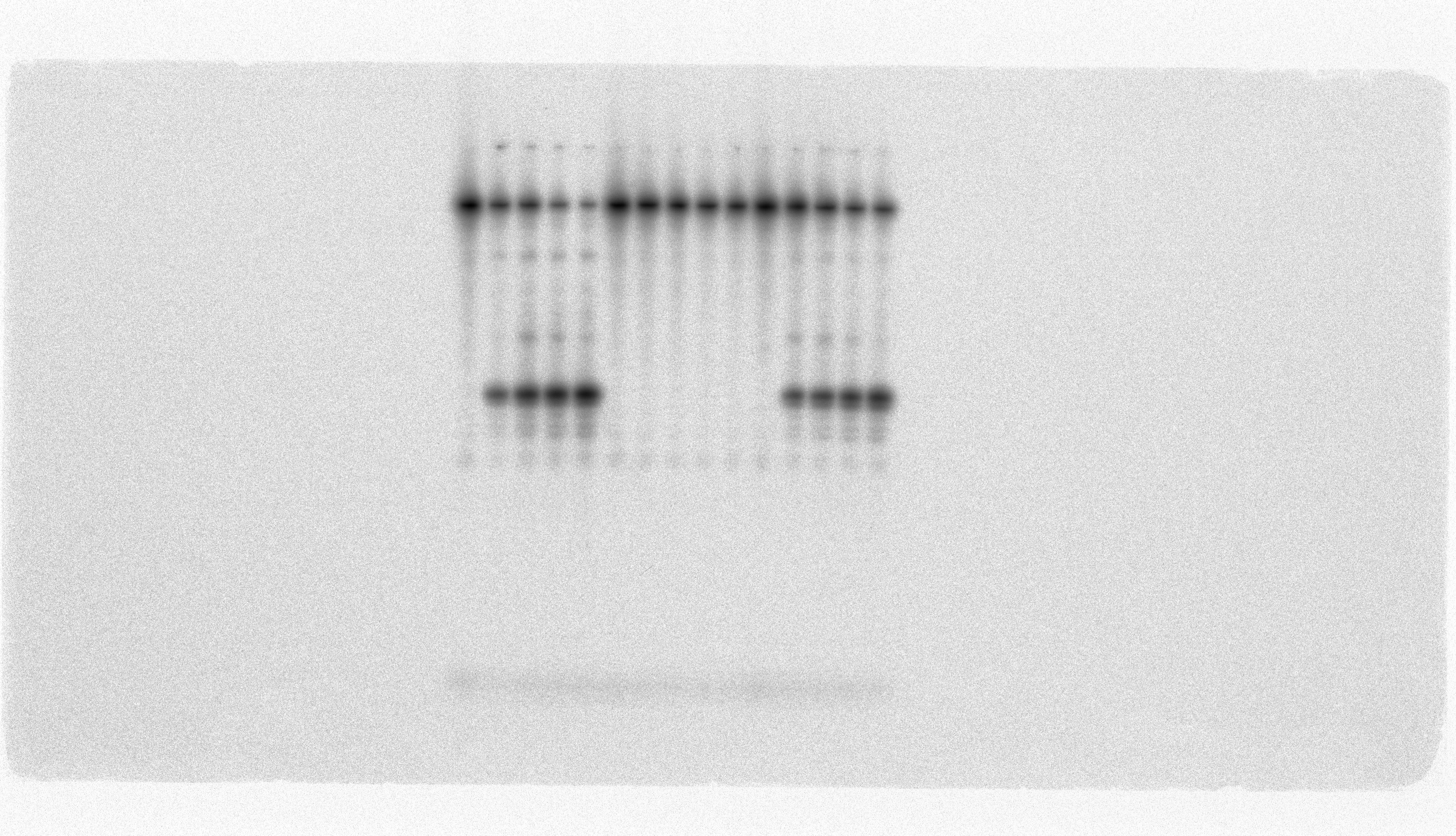

Supplement: Figure 2—source data 1. [file elife-70160-fig2-data1.zip › Figure_2_source-data_1/Aq_wt_d172-191_d184-191_50nM_raw.jpg]

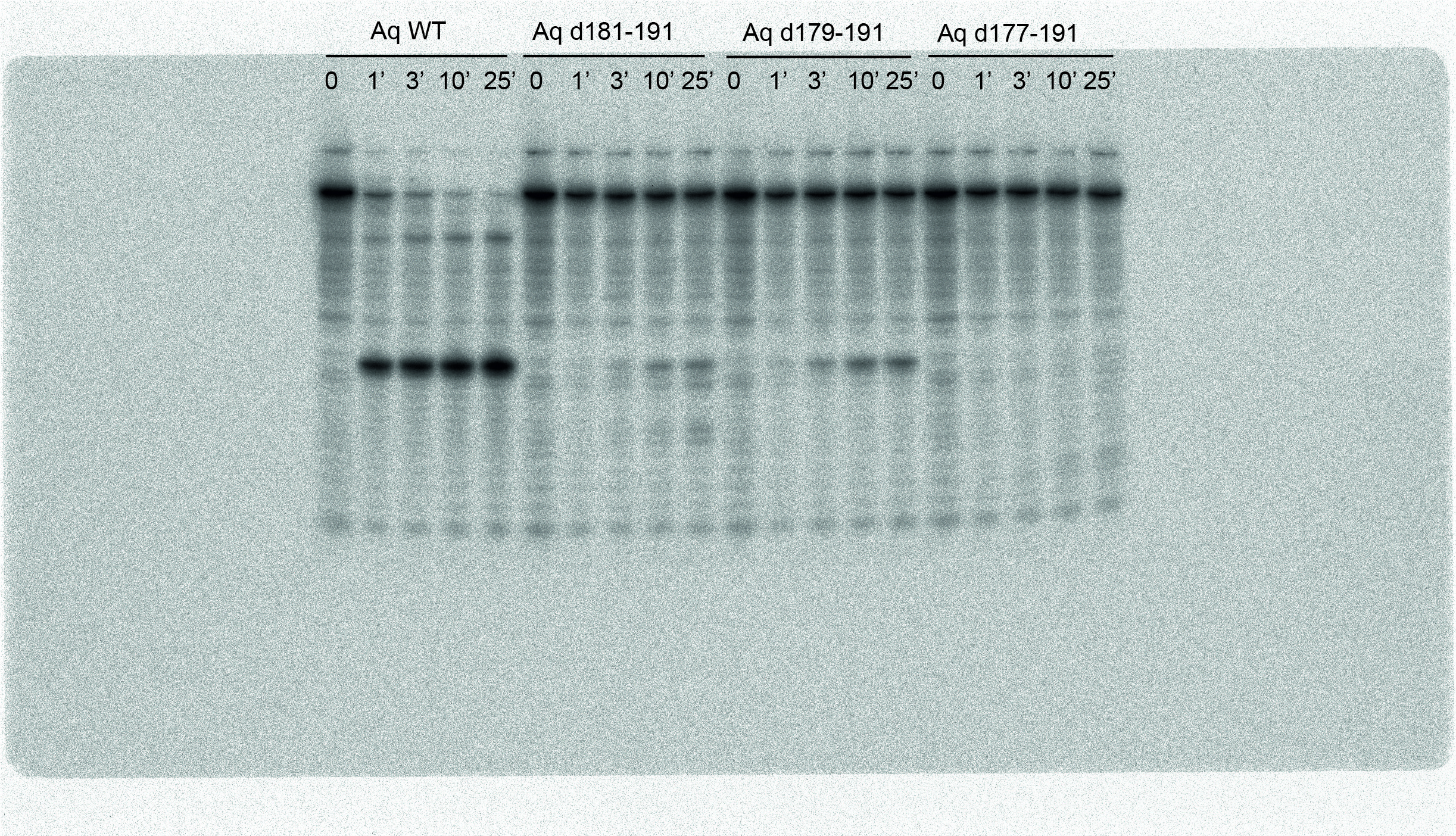

Supplement: Figure 2—source data 1. [file elife-70160-fig2-data1.zip › Figure_2_source-data_1/Aq_wt_d181_179_d177_500nM_labeled.jpg]

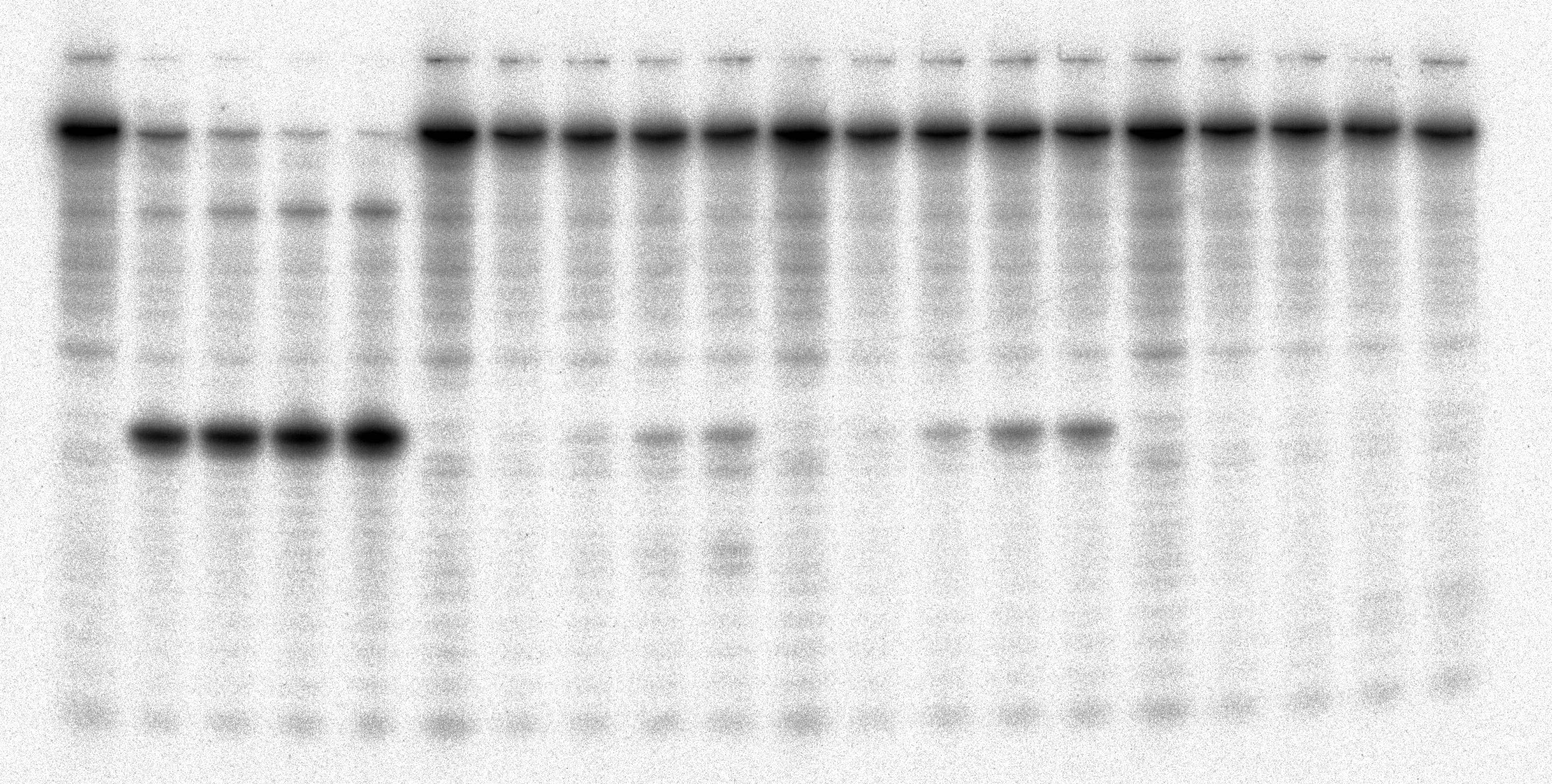

Supplement: Figure 2—source data 1. [file elife-70160-fig2-data1.zip › Figure_2_source-data_1/Aq_wt_d181_179_d177-191_500nM_raw.jpg]

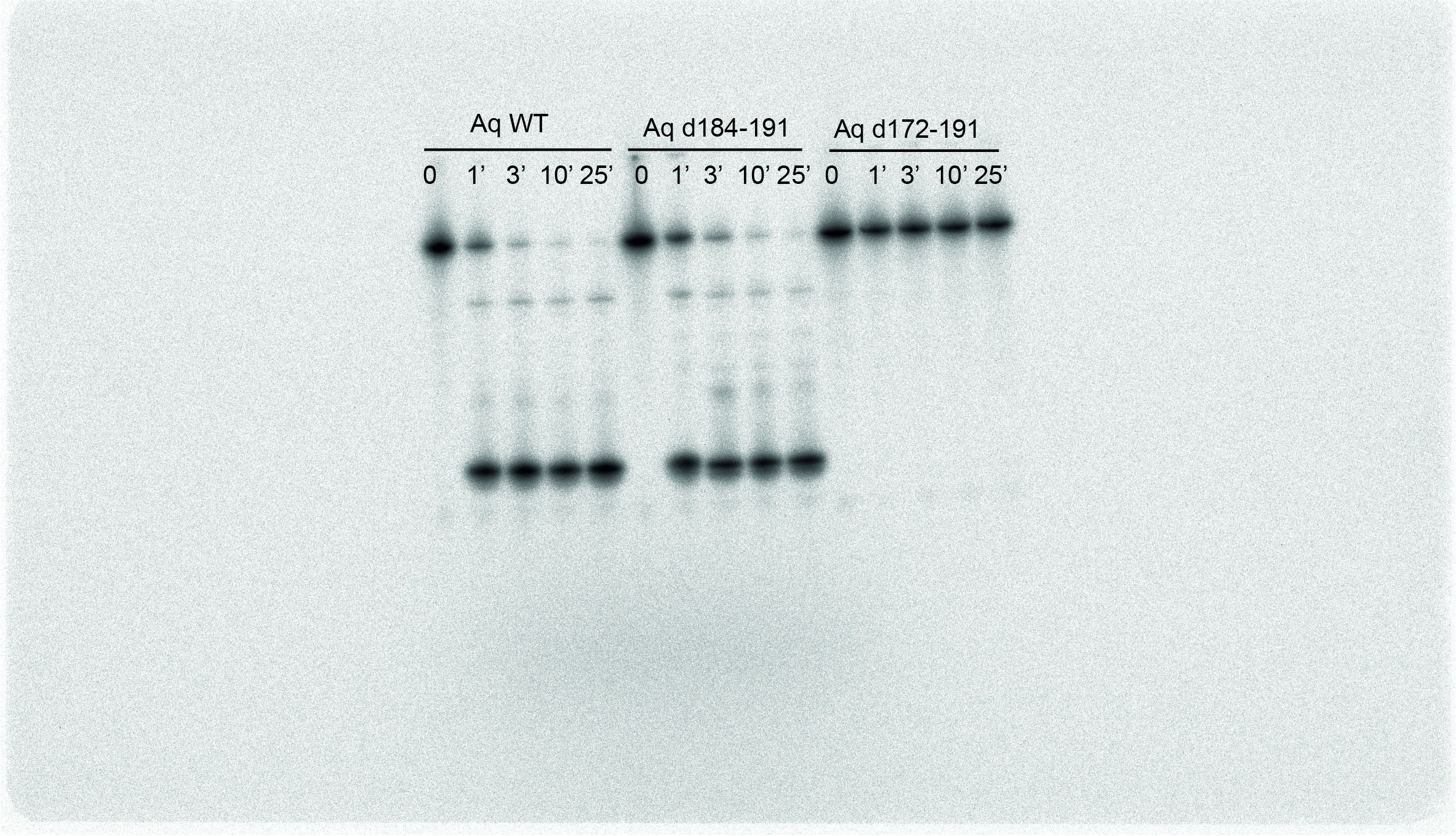

Supplement: Figure 2—source data 1. [file elife-70160-fig2-data1.zip › Figure_2_source-data_1/Aq_wt_d184-191_d172_500nM_labeled.jpg]

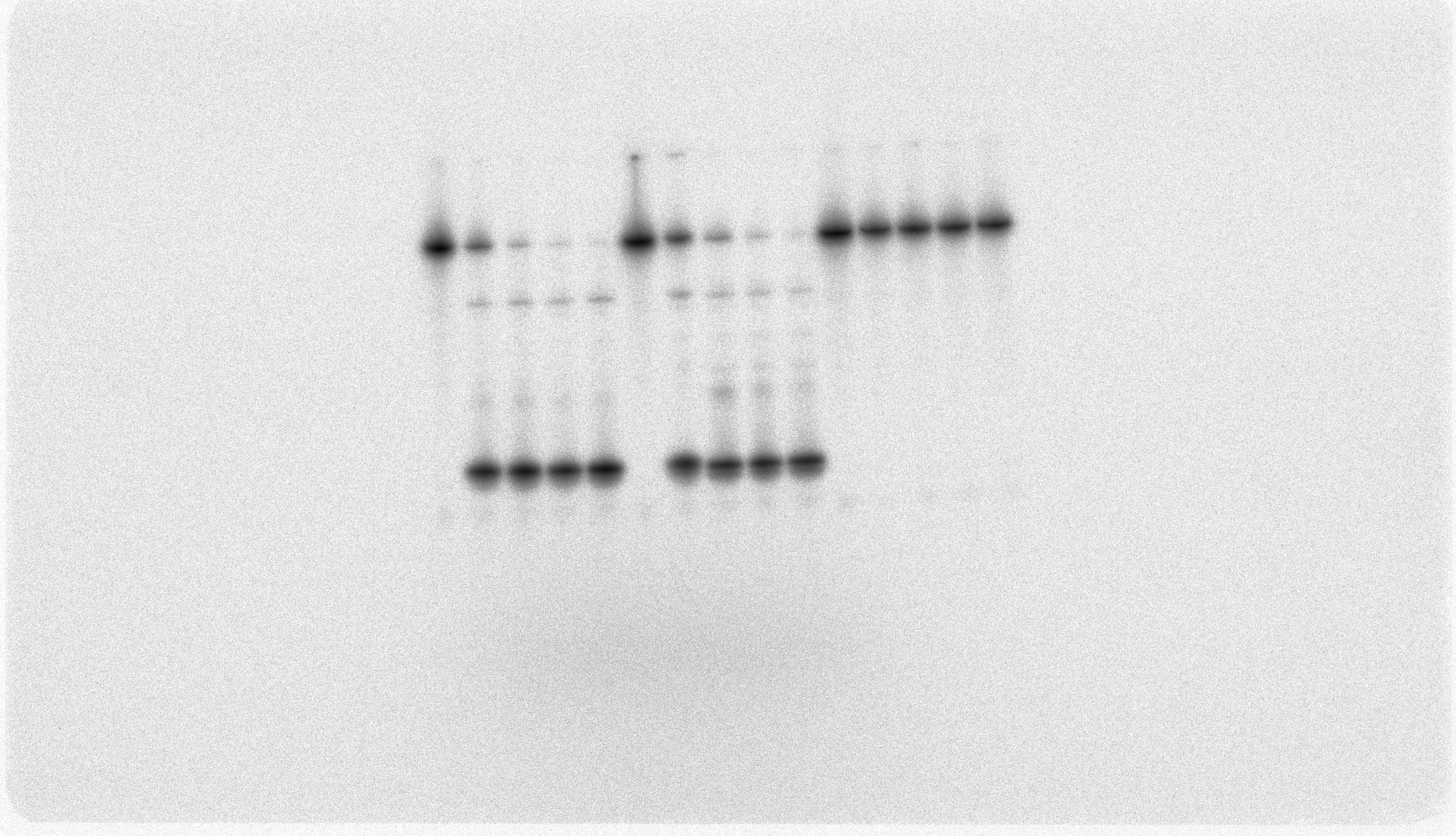

Supplement: Figure 2—source data 1. [file elife-70160-fig2-data1.zip › Figure_2_source-data_1/Aq_wt_d184-191_d172-191_500nM_raw.jpg]

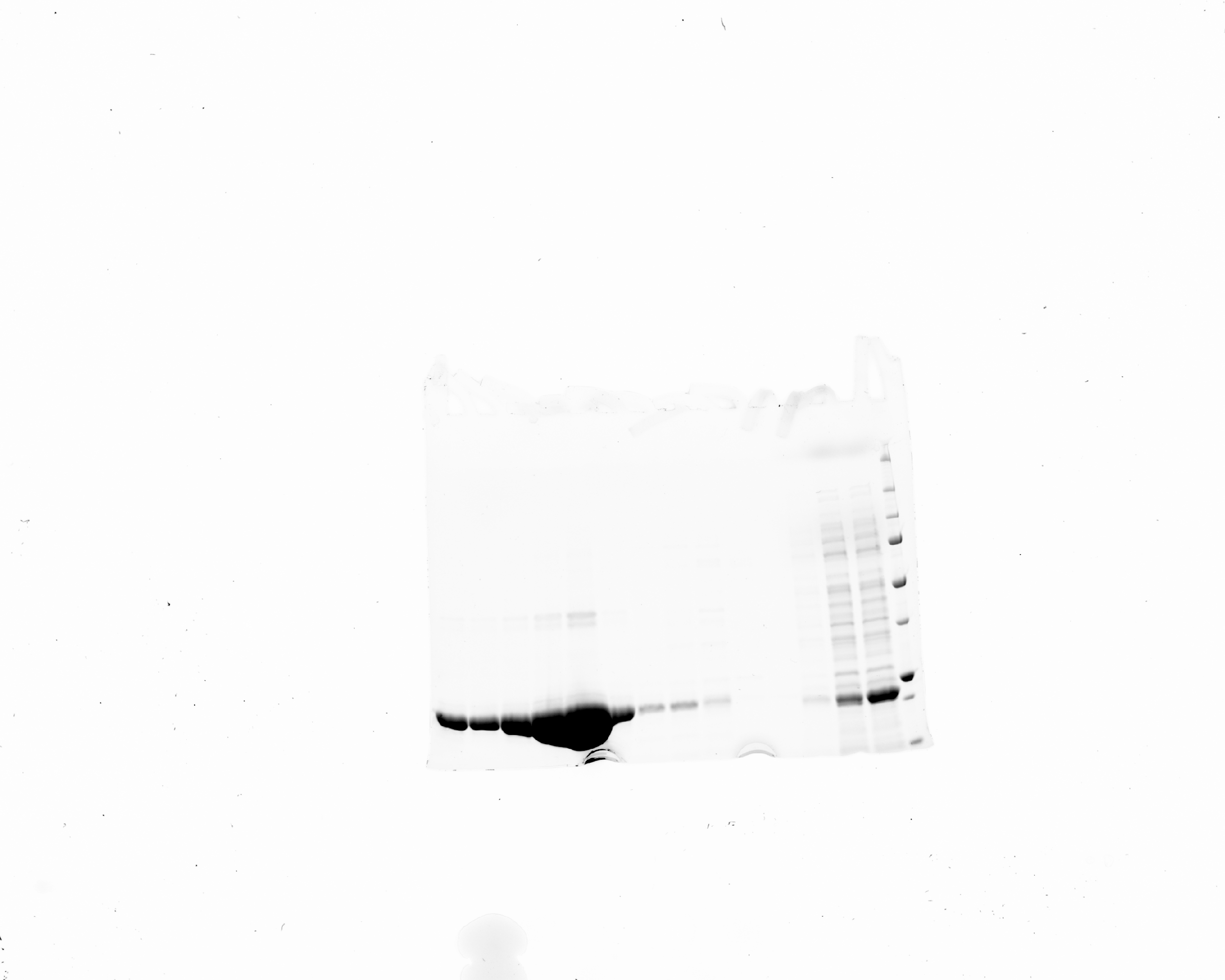

Supplement: Figure 2—figure supplement 1—source data 1. [file elife-70160-fig2-figsupp1-data1.zip › Coomassie_PAGE_HisTrap_Aq880-raw.tif]

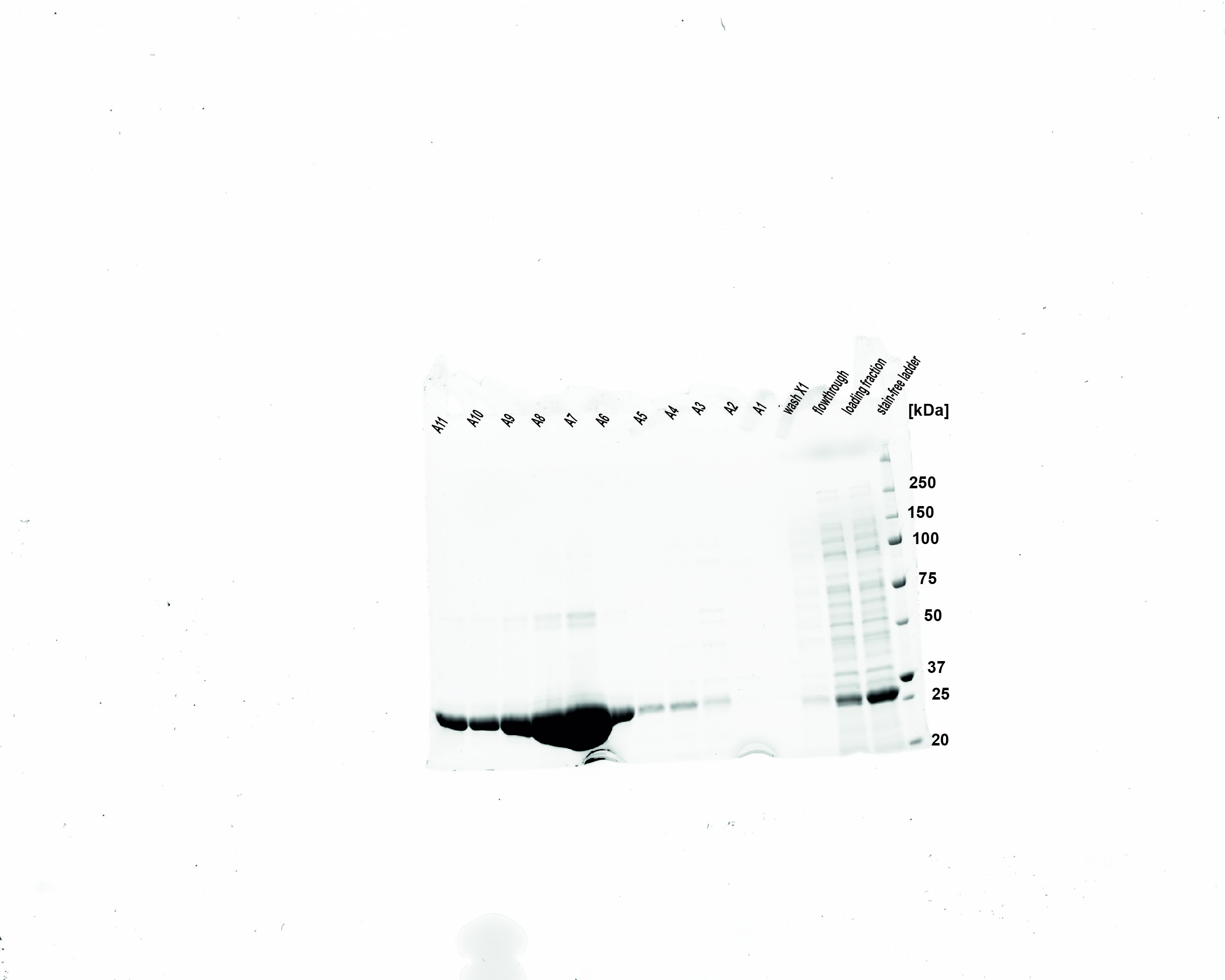

Supplement: Figure 2—figure supplement 1—source data 1. [file elife-70160-fig2-figsupp1-data1.zip › Coomassie_PAGE_HisTrap_Aq880-labeled.jpg]

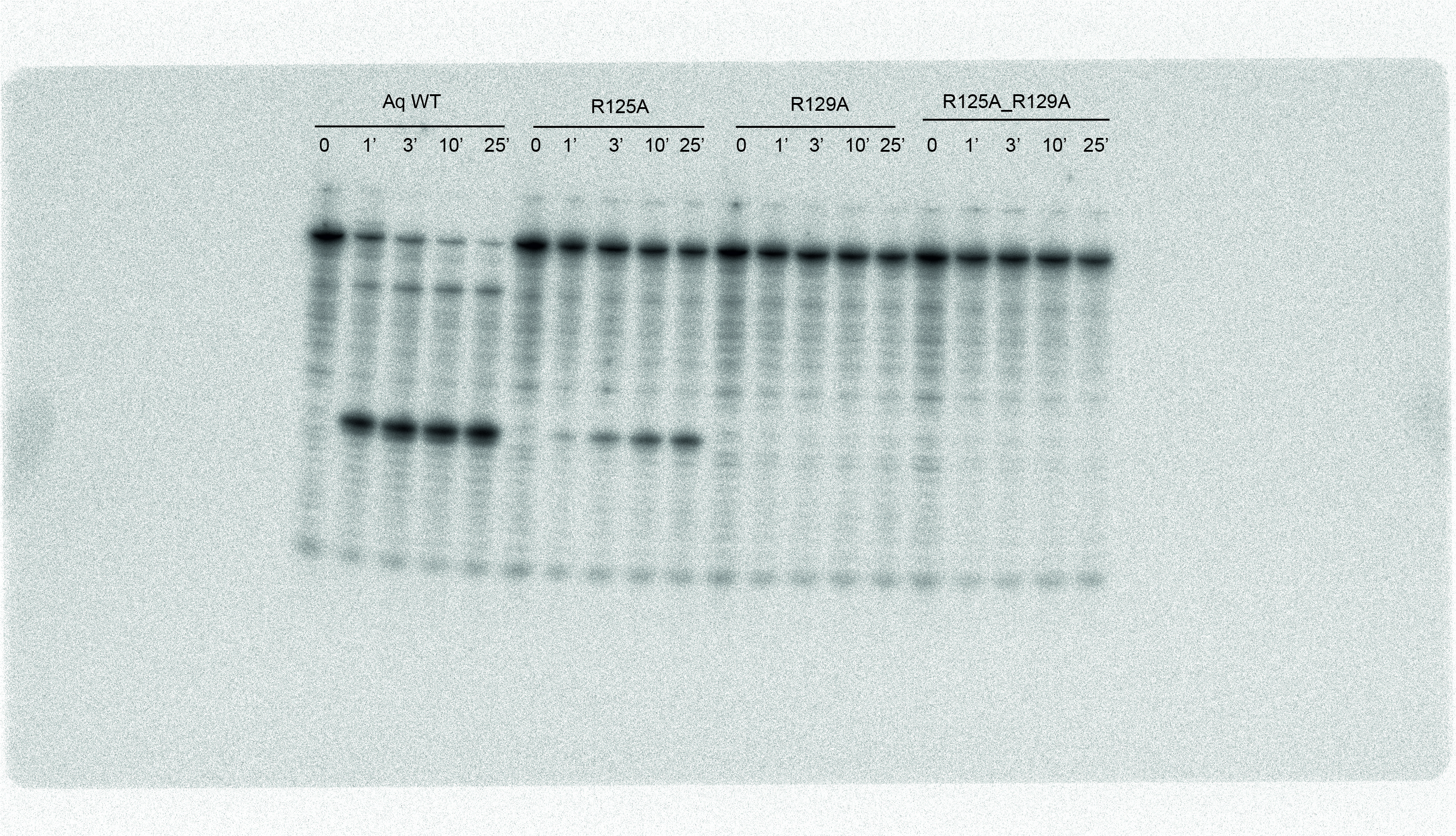

Supplement: Figure 4—source data 1. [file elife-70160-fig4-data1.zip › Figure_4_source_data_1/Aq_wt_R125A_R129A_R125AR129A_500nM_labeled.jpg]

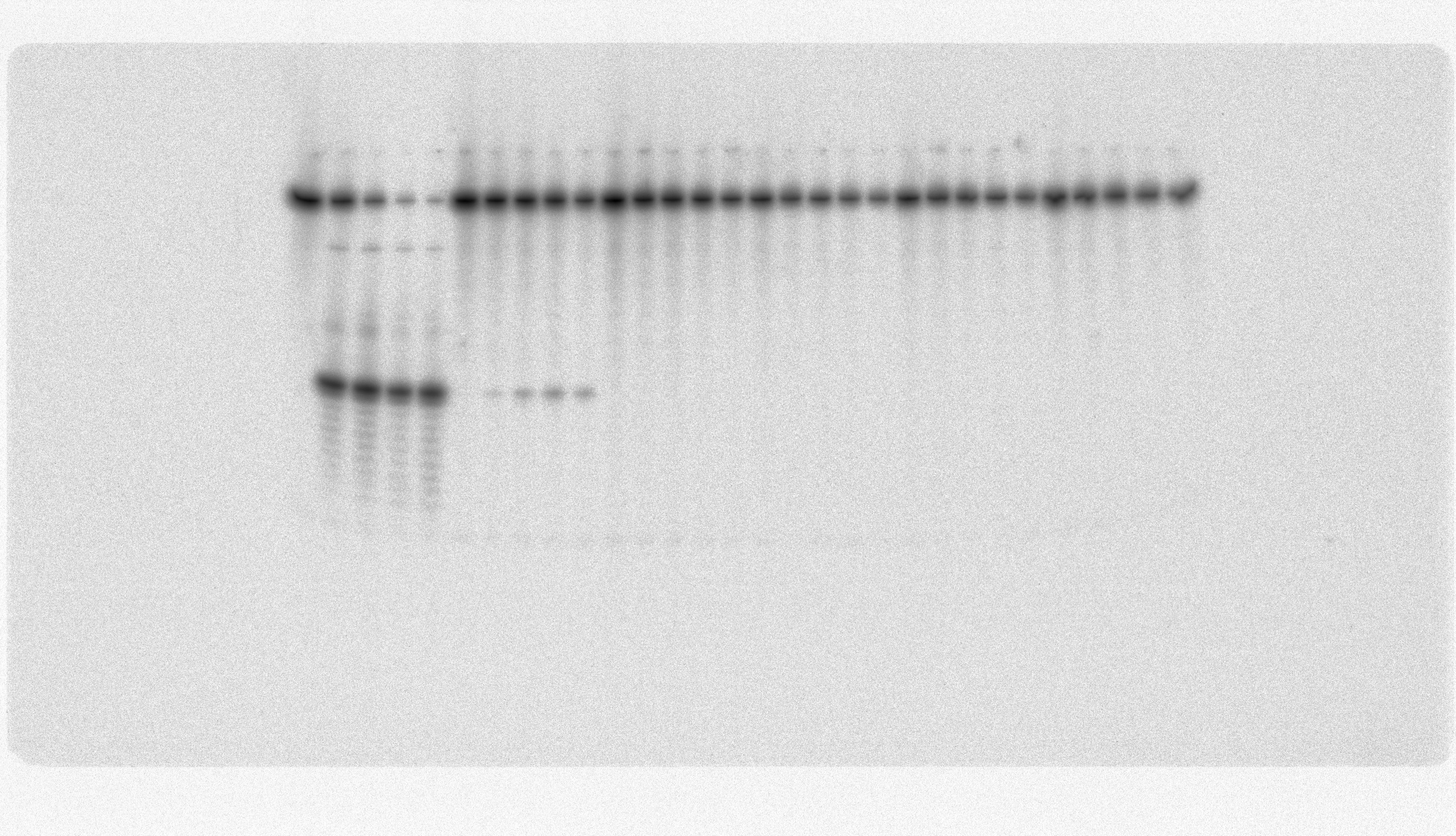

Supplement: Figure 4—source data 1. [file elife-70160-fig4-data1.zip › Figure_4_source_data_1/Aq_wt_R125A_R129A_R125AR129A_50nM_raw.jpg]

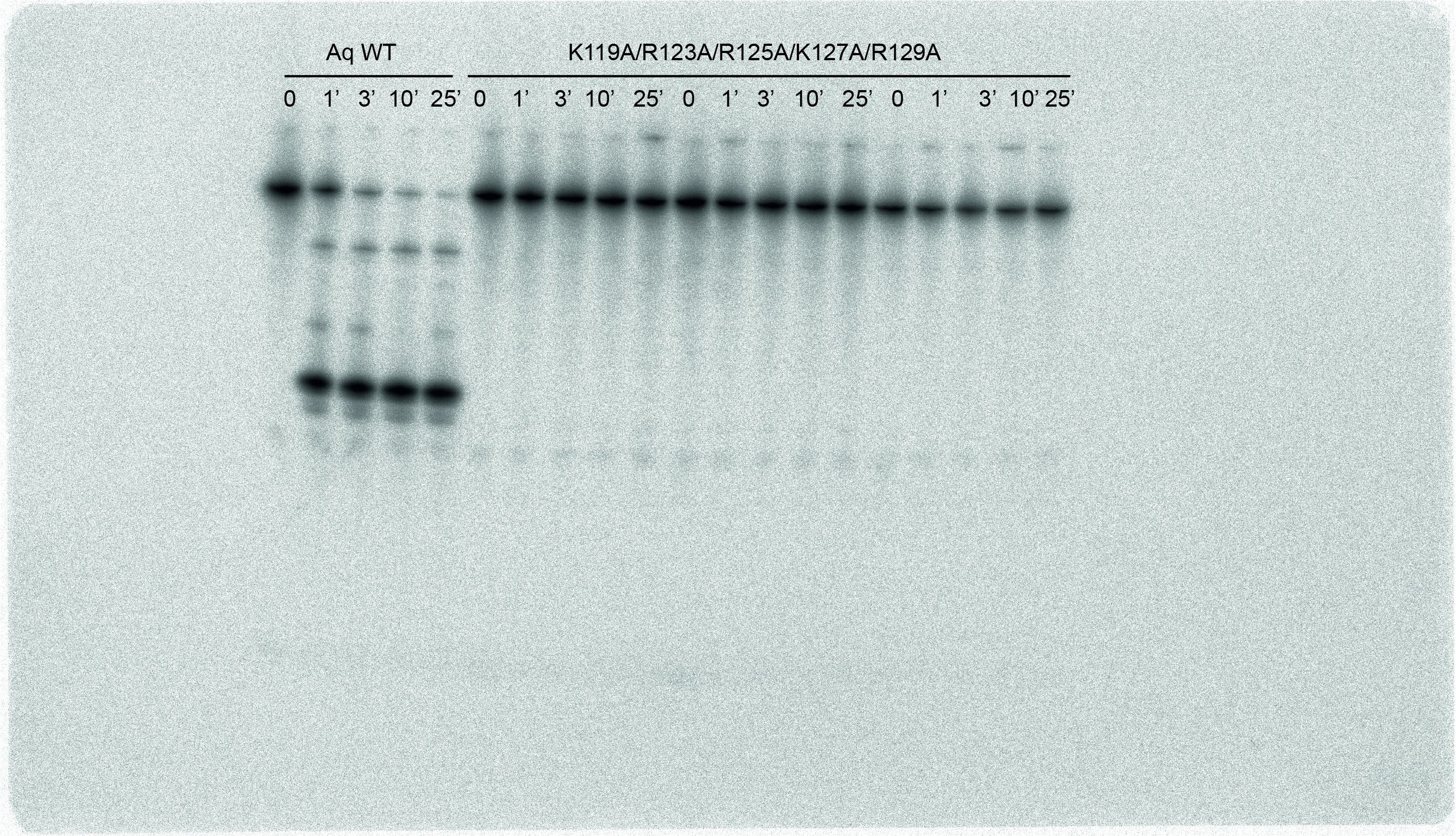

Supplement: Figure 4—source data 1. [file elife-70160-fig4-data1.zip › Figure_4_source_data_1/Aq_wt_5xarginine_500nM_labeled.jpg]

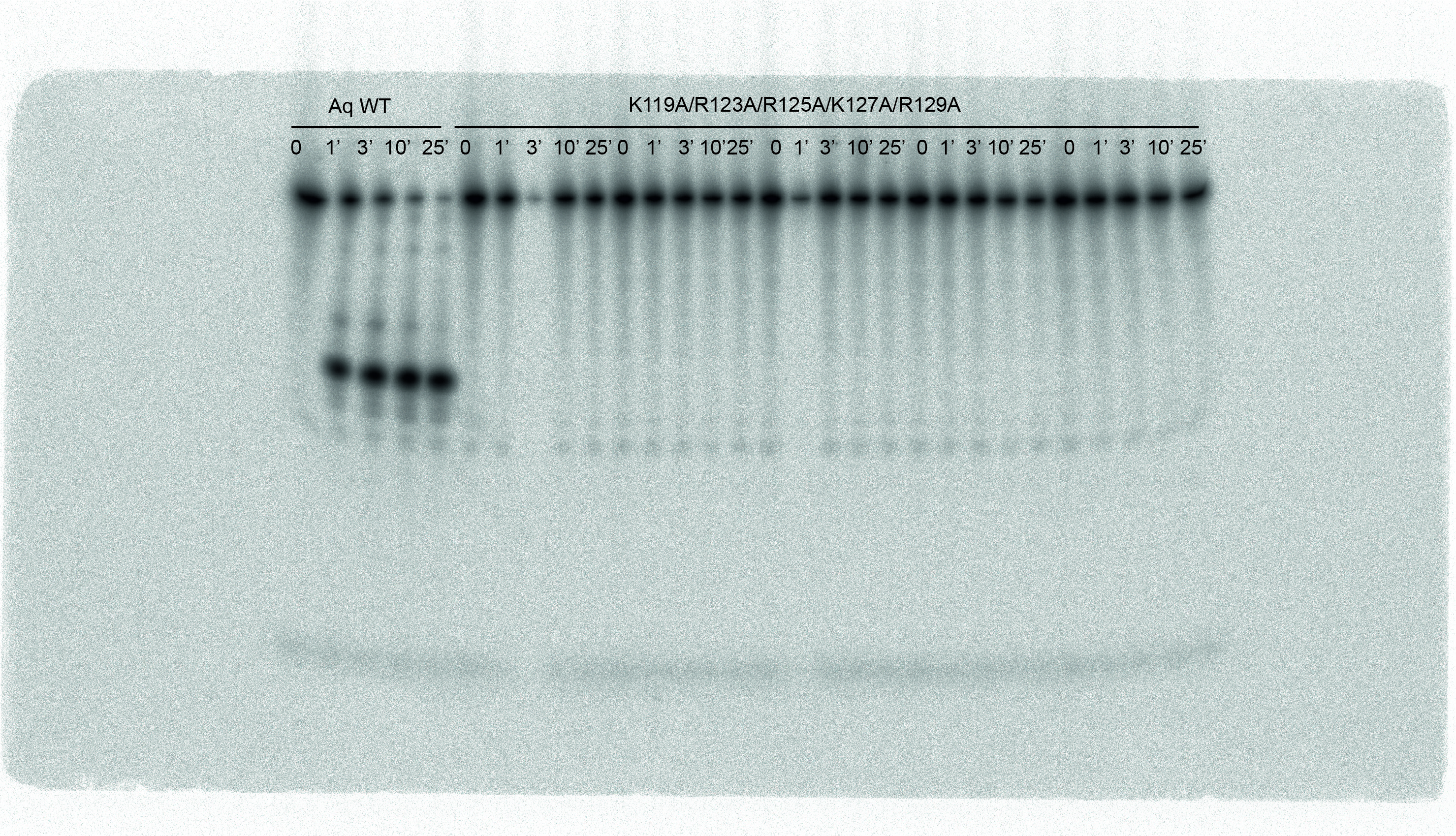

Supplement: Figure 4—source data 1. [file elife-70160-fig4-data1.zip › Figure_4_source_data_1/Aq_wt_5xarginine_50nM_labeled.jpg]

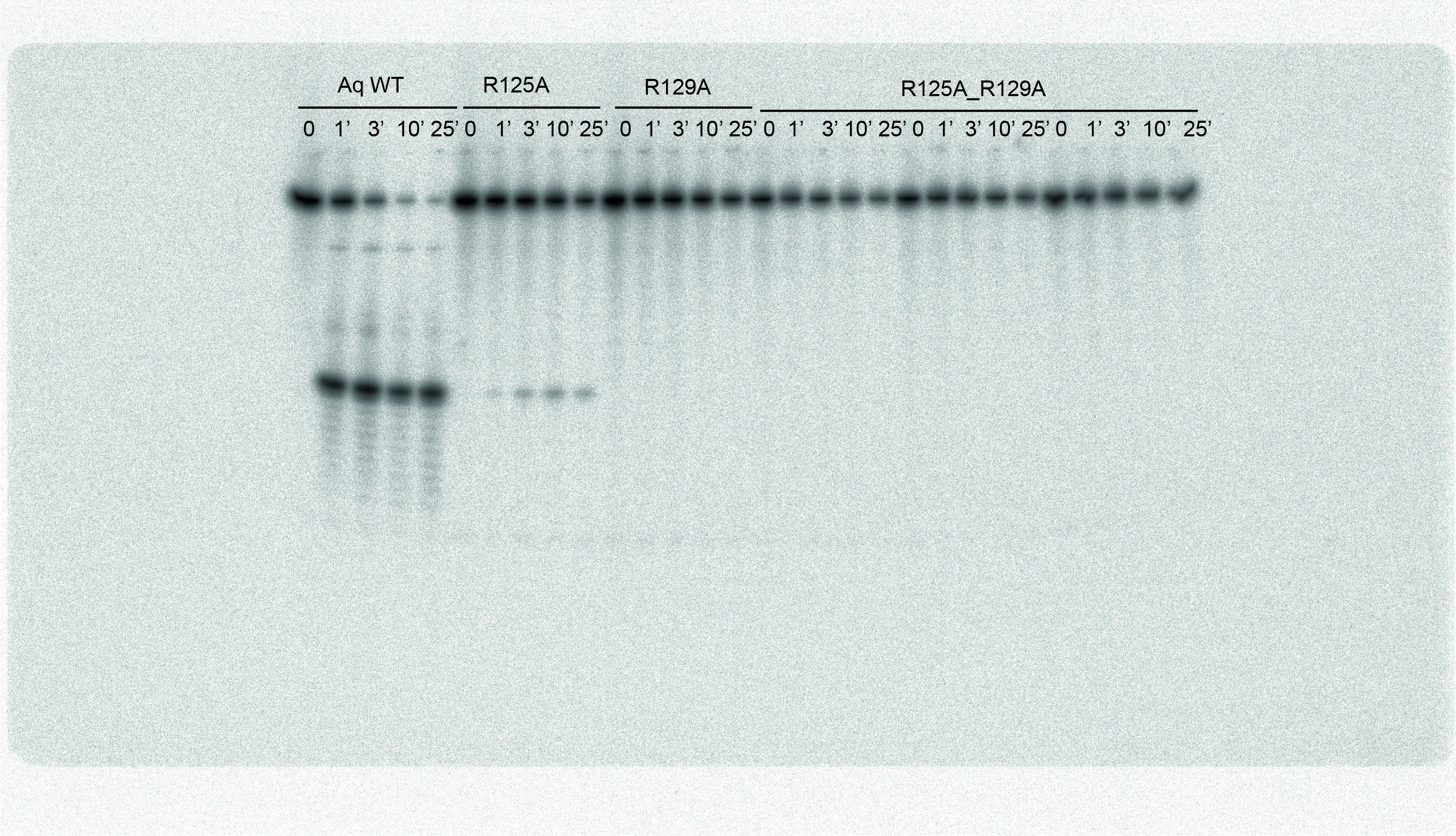

Supplement: Figure 4—source data 1. [file elife-70160-fig4-data1.zip › Figure_4_source_data_1/Aq_wt_R125A_R129A_R125AR129A_50nM_labeled.jpg]

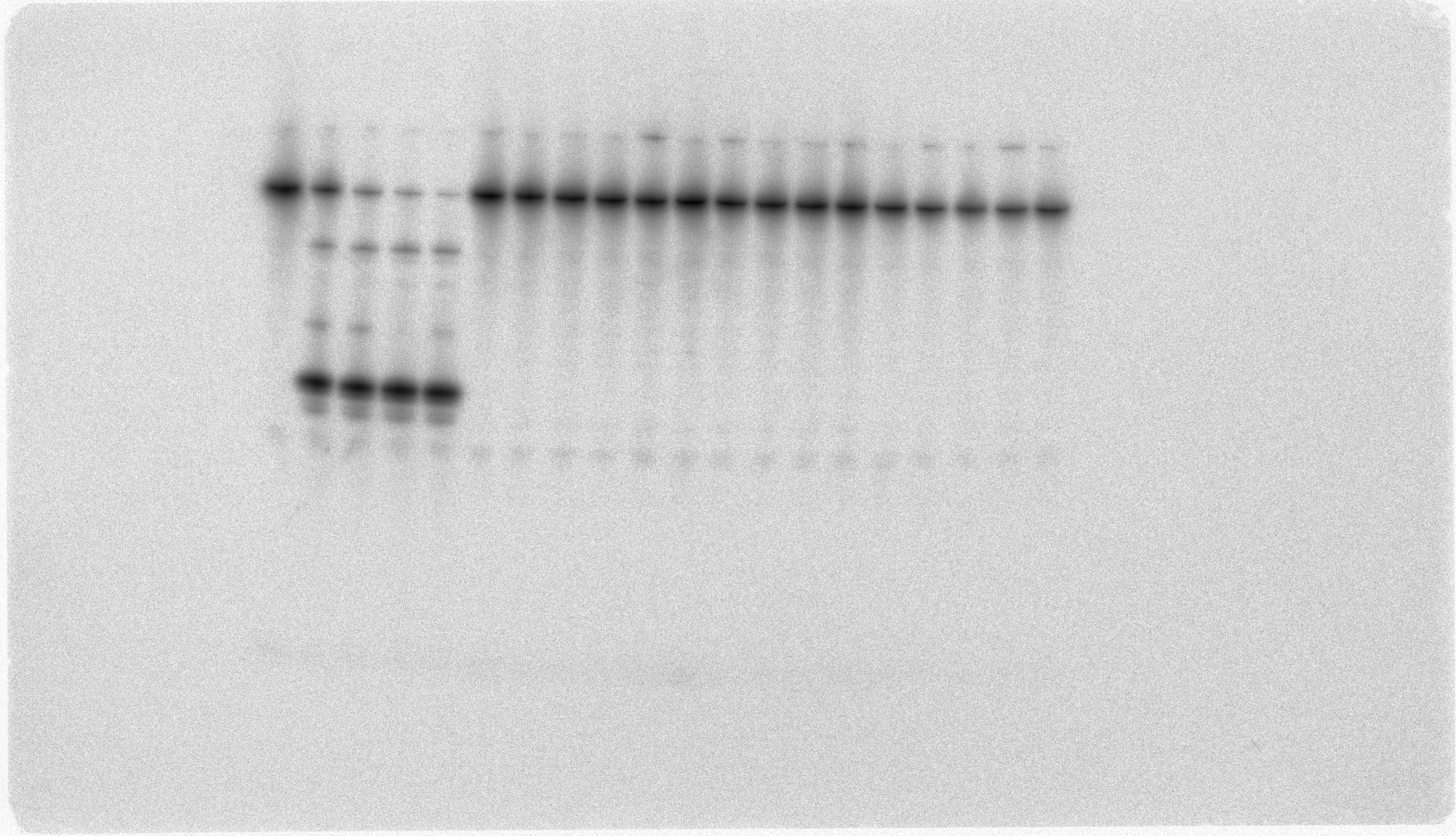

Supplement: Figure 4—source data 1. [file elife-70160-fig4-data1.zip › Figure_4_source_data_1/Aq_wt_5xarginine_500nM_raw.jpg]

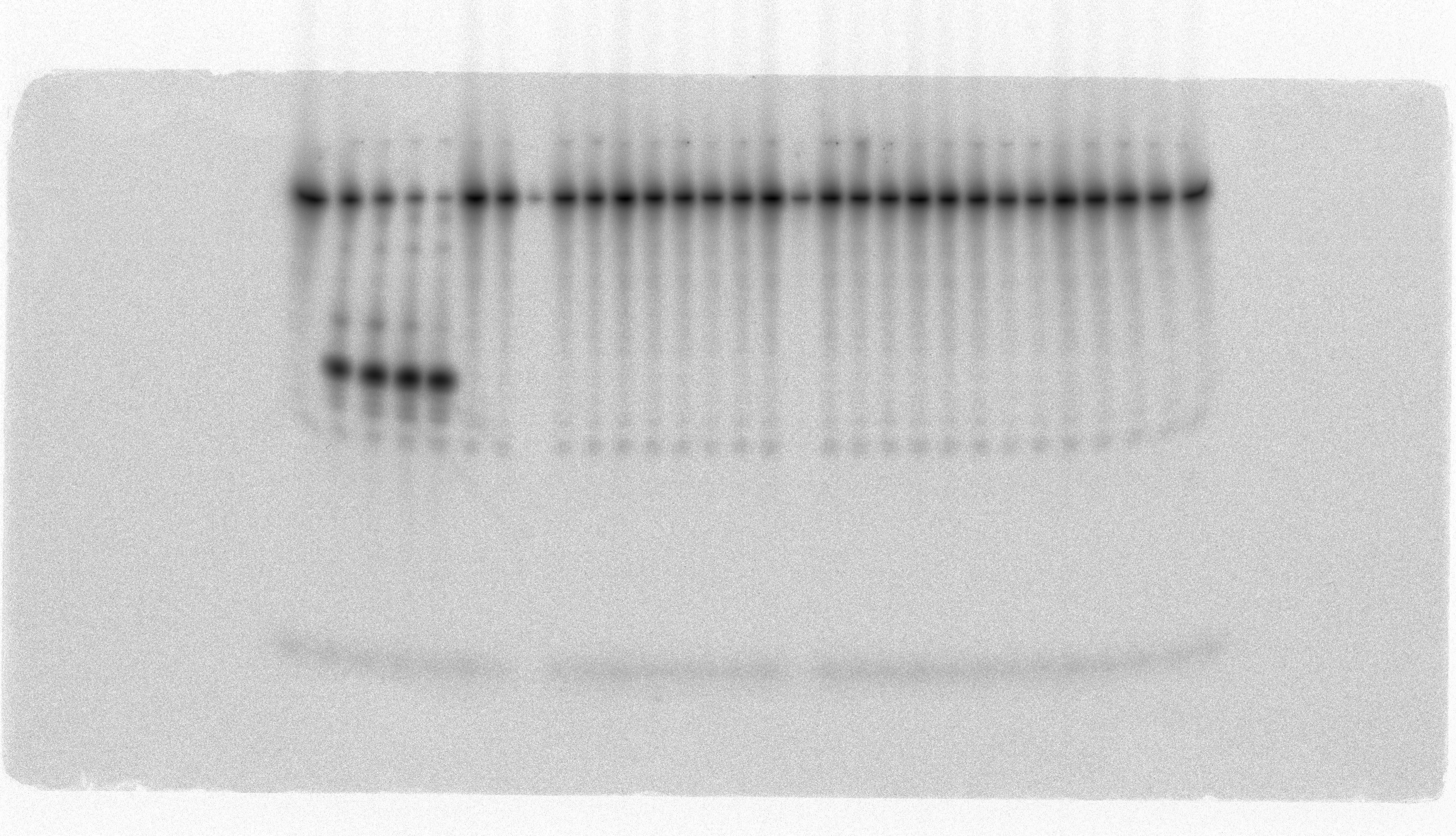

Supplement: Figure 4—source data 1. [file elife-70160-fig4-data1.zip › Figure_4_source_data_1/Aq_wt_5xarginine_50nM_raw.jpg]

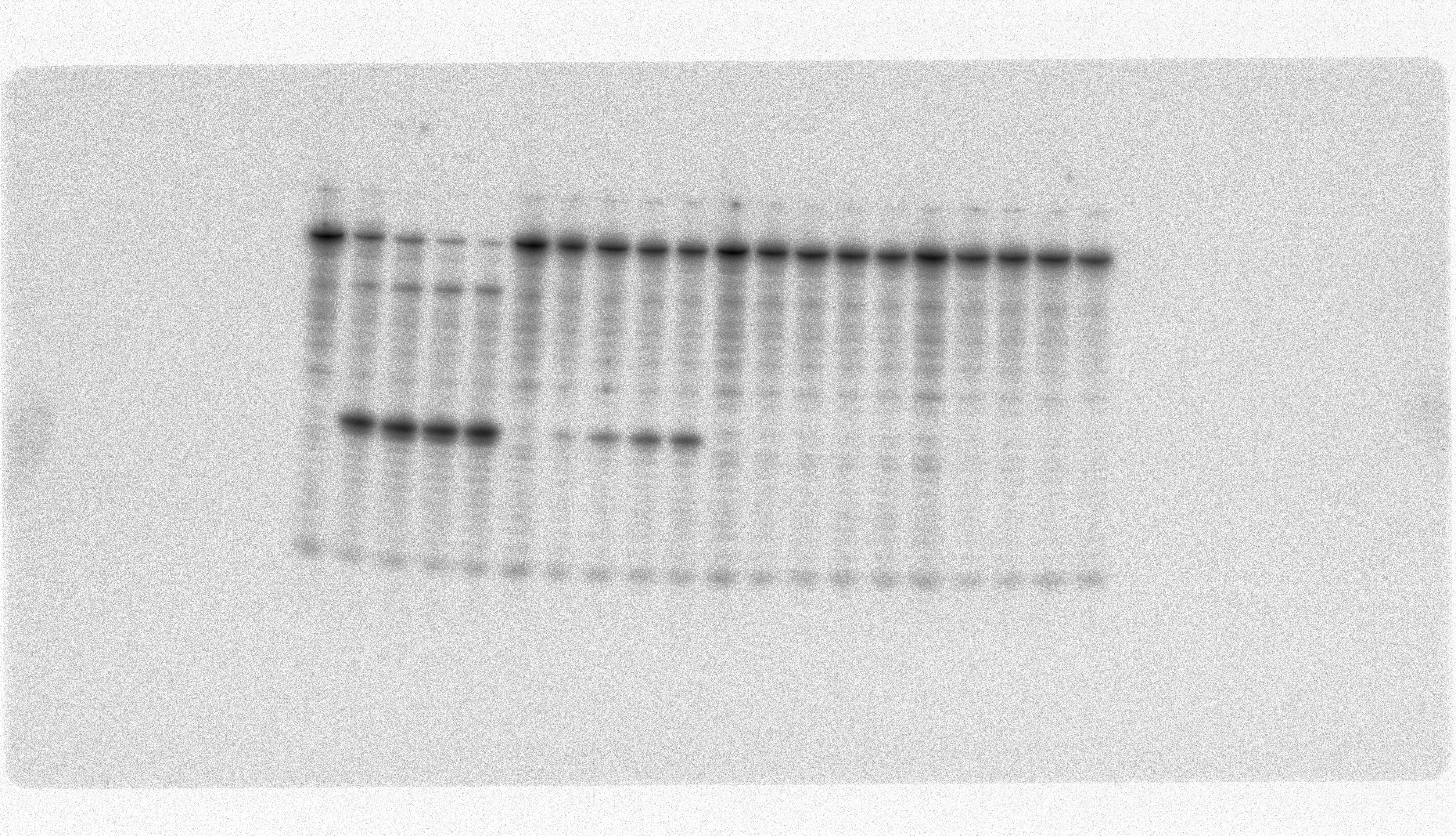

Supplement: Figure 4—source data 1. [file elife-70160-fig4-data1.zip › Figure_4_source_data_1/Aq_wt_R125A_R129A_R125AR129A_500nM_raw.jpg]
