# Supplementary material for: Structure and mechanistic features of the prokaryotic minimal RNase P
Source: eLife. 2021 Jun 28;10:e70160. doi: 10.7554/eLife.70160 (PMC8266387; doi:10.7554/eLife.70160)
Supplement: Figure 1—figure supplement 2—source data 1. [file elife-70160-fig1-figsupp2-data1.zip › Figure 1-figure supplement 2-source data 1/Figure 1-figure supplement 2-source data 1.docx]

**Figure S2 -source data 1**

**RNase P processing of 5'-^32^P-labeled *T. thermophilus* pre-tRNA^Gly^ by Aq880 and Hhal2243**

This zip archive contains the raw phosphor images shown in figure S2 with and without the respective label as indicated by “raw” and “labeled” in the file name.
