# Supplementary material for: Structure and mechanistic features of the prokaryotic minimal RNase P
Source: eLife. 2021 Jun 28;10:e70160. doi: 10.7554/eLife.70160 (PMC8266387; doi:10.7554/eLife.70160)
Supplement: Figure 4—source data 1. [file elife-70160-fig4-data1.zip › Figure_4_source_data_1/Figure 4-source data 1.docx]

**Figure 3 -source data 1**

**Processing of pre-tRNA^Gly^ by Aq880 wt and derived arginine and lysine to alanine variants.**

This zip archive contains the raw phosphor images shown in figure 3 with and without the respective label as indicated by “raw” and “labeled” in the file name.
