# Supplementary material for: Structure and mechanistic features of the prokaryotic minimal RNase P
Source: eLife. 2021 Jun 28;10:e70160. doi: 10.7554/eLife.70160 (PMC8266387; doi:10.7554/eLife.70160)
Supplement: Supplementary file 1. [file elife-70160-supp1.docx]

**Supplementary File 1**

**Structure and mechanistic features of the prokaryotic minimal RNase P**

Rebecca Feyh^1,§^, Nadine B. Wäber^1,§^, Simone Prinz^2^, Pietro Ivan Giammarinaro^3^, Gert Bange^3,4^, Georg Hochberg^4^, Roland K. Hartmann^1*^ and Florian Altegoer^3*^

^1^Institute of Pharmaceutical Chemistry, Philipps-University Marburg, 35037 Marburg, Germany

^2^Department of Structural Biology, Max Planck Institute of Biophysics, Frankfurt, Germany

^3^Center for Synthetic Microbiology and Department of Chemistry, Philipps-University Marburg, 35043 Marburg, Germany

^4^Max-Planck Institute for terrestrial Microbiology, Max-von-Frisch-Straße 10, Marburg, Germany

§ joint first authorship

*to whom correspondence should be addressed: Florian Altegoer (altegoer@uni-marburg.de) or Roland K. Hartmann (hartmanr@staff.uni-marburg.de)

**Keywords:** *Aquifex aeolicus* RNase P, HARP, Cryo-EM, mass photometry

**Supplementary File 1a. Cryo-EM data collection, refinement and validation statistics.**

|  | Hhal2243  (EMD-12878)  (PDB: 7OG5) |
| --- | --- |
| **Data collection and processing** |  |
| Magnification | 105,000 |
| Voltage (kV) | 300 |
| Electron exposure (e-/Å^2^) | 40 |
| Defocus range (µm) | 1.5 – 2.4 |
| Pixel size (Å) | 0.833 |
| Symmetry imposed | C1 |
| Initial particle images (no.) | 2,749,587 |
| Final particle images (no.) | 1,736,597 |
| Map Resolution (Å) | 3.37 |
| FSC threshold | 0.143 |
| Map resolution range | 3.37 – 5 Å |
|  |  |
| **Refinement** |  |
| Map sharpening *B* factor (Å^2^) | 181 |
| Model composition |  |
| Non-hydrogen atoms | 15444 |
| Protein residues | 1892 |
| Ligands | 0 |
| *B* factors (Å^2^) |  |
| Protein | 106.70 |
| Ligand | 0 |
| R.m.s. deviations |  |
| Bond Lengths (Å) | 0.01 |
| Bond angles (°) | 0.933 |
| Validation |  |
| MolProbity score | 2.33 |
| Clashscore | 19.16 |
| Poor rotamers (%)  Ramachandran plot | 0 |
| Favored (%)  Allowed (%)  Outliers (%) | 89.91  10.09  0.00 |

**Supplementary File 1b**: Dynamic mass distribution determined by mass photometry

| **Sample** | **Peak** | **Molecular weight (kDa)** | **Amount (%)** |
| --- | --- | --- | --- |
| **Hhal2243 wt** | 1 | 295 | 88 |
| **Aq880 wt** | 1 | 42 | 7 |
|  | 2 | 78 | 13 |
|  | 3 | 129 | 9 |
|  | 4 | 182 | 13 |
|  | 5 | 234 | 11 |
|  | 6 | 277 | 47 |
| **Aq880_Δ184-191** | 1  2  3  4  5 | 42  83  133  183  235 | 32  50  12  4  2 |
| **Aq880_Δ181-191** | 1 | 42 | 55 |
|  | 2 | 81 | 41 |
|  | 3 | 134 | 4 |
| **Aq880_Δ179-191** | 1 | 44 | 90 |
| **Aq880_Δ177-191** | 1 | 52 | 92 |
| **Aq880_R125A** | 1  2  3  4  5 | 85  134  181  246  284 | 3  5  6  15  59 |
| **Aq880_R129A** | 1  2  3  4  5 | 71  135  185  247  286 | 3  6  7  17  58 |
| **Aq880_ K119A/R123A/ R125A/K127A/R129A** | 1  2  3  4  5 | 94  143  193  243  278 | 3  4  9  19  58 |

**Supplementary File 1c**: **Primers & Plasmids used in this study**

| **constructs in pET-28a(+)** | **sequence (5‘🡪 3‘)** |
| --- | --- |
| aq880 | AAG CCA TGG ATG TGT TCG TTC TCG ACA C &  CTC TCG AGA AAC CTG TGT CTT ACC AAG C |
| aq880_∆184-191 | TTT CTC GAG CAC CAC CAC CAC CAC C &  AATGTTTTTGAAATTCTTAGGGTCTATG |
| aq880_∆181-191 | TTT CTC GAG CAC CAC CAC CAC CAC C &  GAA ATT CTT AGG GTC TAT GAG TTT TAT ACC |
| aq880_∆179-191 | TTT CTC GAG CAC CAC CAC CAC CAC C &  CTT AGG GTC TAT GAG TTT TAT ACC TAT C |
| aq880_∆177-191 | TTT CTC GAG CAC CAC CAC CAC CAC C &  GTC TAT GAG TTT TAT ACC TAT CTT GTC CG |
| aq880_∆172-191 | TTT CTC GAG CAC CAC CAC CAC CAC C &  TAT CTT GTC CGC CCA TGT TCT GAG GCC |
| aq880_K119A/R123A/ R125A/K127A/R129A | GCG CAG CTA TTA ATG CCC CGA CGT CTT CAC &  TCG CGG AGG CGT ACG CGG AAG CCC TCA GG |
| aq880_R125A/R129A | GCC TAG CTA TTA ATT TCC CGA CGT CTT CAC &  TCG CGG AGA AGT ACG CGG AAG CCC TCA GG |
| aq880_R125A | GCC TAG CTA TTA ATT TCC CGA CGT CTT CAC &  TCG CGG AGA AGT ACA GGG AAG CCC TCA GG |
| aq880_R129A | GCC TAG CTA TTA ATT TCC CGA CGT CTT CAC &  TCA GGG AGA AGT ACG CGG AAG CC |
| hhal2243 | TTT GCT AGC CGC CGA TTC GTG CTC G &  TTT GCT CAG CCT ACC CGG CGG GCT G |

**Supplementary References**

1. Nickel AI, Wäber NB, Gößringer M, Lechner M, Linne U, Toth U, Rossmanith W, Hartmann RK (2017) Minimal and RNA-free RNase P in Aquifex aeolicus. *Proc Natl Acad Sci U S A* 114(42):11121–11126.

2. Schwarz TS, Wäber NB, Feyh R, Weidenbach K, Schmitz RA, Marchfelder A, Hartmann RK (2019) Homologs of aquifex aeolicus protein-only RNase P are not the major RNase P activities in the archaea haloferax volcanii and methanosarcina mazei. *IUBMB Life* 71(8):1109–1116.

3. Buchan DWA, Jones DT (2019) The PSIPRED Protein Analysis Workbench: 20 years on. *Nucleic Acids Res* 47(W1):W402–W407.
